# Small RNAs: an ideal choice as sperm quality biomarkers

**DOI:** 10.3389/frph.2024.1329760

**Published:** 2024-02-09

**Authors:** Poonam Mehta, Rajender Singh

**Affiliations:** ^1^ Division of Endocrinology, Central Drug Research Institute, Lucknow, India; ^2^ Division of Endocrinology, Academy of Scientific and Industrial Research (AcSIR), Ghaziabad, India

**Keywords:** sperm RNAs, fertility biomarkers, sperm quality, transgenerational inheritance, sperm fertility, small RNAs, sncRNAs

## Abstract

Spermatozoa were classically known as vehicles for the delivery of the paternal genome to the oocyte. However, in 1962, spermatozoa were discovered to carry significant amounts of RNA in them, which raised questions about the significance of these molecules in such a highly specialized cell. Scientific research in the last six decades has investigated the biological significance of sperm RNAs by various means. Irrespective of what sperm RNAs do, their presence in spermatozoa has attracted attention for their exploitation as biomarkers of fertility. Research in this direction started in the year 2000 and is still underway. A major hurdle in this research is the definition of the standard human sperm RNAome. Only a few normozoospermic samples have been analyzed to define the normal sperm RNAome. In this article, we provide a perspective on the suitability of sperm RNAs as biomarkers of fertility and the importance of defining the normal sperm RNAome before we can succeed in identifying RNA-based biomarkers of sperm quality and fertility. The identification of sperm RNA biomarkers of fertility can be exploited for quality screening of donor sperm samples, explain infertility in idiopathic cases, and RNA therapeutics for the treatment of male infertility.

## 1 Introduction

Spermatozoa were thought to be the vehicles for the delivery of the paternal genome to the oocyte. This miniature and the only actively motile cell in the human body prepares itself for its journey in a unique manner, which requires packaging the paternal genome tightly to safely deliver it to the oocyte. Over a period of time, it was realized that spermatozoa carry a plethora of RNAs that are diverse in nature. The first evidence of RNAs in spermatozoa was presented by Indian scientists at the Centre for Cellular and Molecular Biology, Hyderabad, formerly the Regional Research Laboratory, Hyderabad, India (1). Further investigations on sperm RNAs showed that spermatozoa are unusually rich in small RNAs in comparison to coding RNAs (2). Transcription was already proven to be shut off in spermatozoa, leaving no possibility of the production of new RNAs (3). This meant that RNAs stored in spermatozoa were produced earlier and packaged in the cell. Investigators at that time also proposed that sperm RNAs might be the remnants of past activity, perhaps with no further biological functions. It was suggested that a number of these RNAs have served key roles in the past and are no longer required. Further investigations in this field revealed sperm sncRNAs to undergo an epic journey of gain and loss during epididymal transition (4), which highlighted their biological significance. The massive transformation of sperm RNAs in the epididymis further heightens the curiosity regarding their roles.

## 2 Massive makeover of sperm RNAs

The presence of a particular pattern in sperm RNAs suggests that they are not randomly leftover RNAs; instead, they are biologically selected RNAs that get packaged in spermatozoa. Sperm RNAs also undergo an epic journey of gain and loss during their epididymal transit that remodels the mature sperm small RNA profile. Why a significant remodelling of sperm RNAs is left to an organ beyond the primary reproductive organ (testis) remains an interesting question. Studies focussing on transgenerational inheritance and exposure to adverse environmental conditions suggest that the epididymis offers a window of plasticity in males to adapt to the environment in a constant endeavour to inculcate adaptive changes for improved survival of a species. Another important question to address is whether sperm-borne RNAs are the key to initiating post-fertilization development. The concept of the events in post-fertilization development is beautifully depicted in an article on consolidation and confrontation, which states that the molecular events upon fertilization include molecular interactions to check the compatibility of the gametes before they can proceed for further development (5). According to this concept, both the gametes (sperm and oocyte) have their own set of epigenomes and small RNAs. Upon fertilization, the oocyte epigenome and small RNAs perform a check on the sperm's epigenome and RNAs and vice versa. Sperm that pass this cytoplasmic and genetic compatibility check can proceed with syngamy and further post-fertilization development (5). Incompatibilities at this level can lead to the cessation of embryonic genome activation and further development even after successful fertilization (5). With the first report on the functions of sperm RNAs in post-fertilization development, interest in sperm RNAs grew even further (6). Eventually, a number of these RNAs were shown to be important for early embryonic development (7). In addition to this, evidence from DICER and DROSHA knockout sperm and individual miRNAs have shown that sperm-borne RNAs play critical roles in post-fertilization development (6, 8).

## 3 Sperm RNAs as fertility biomarkers

Sperm RNAs might have served key roles in the testis or may be critical to post-fertilization development. Irrespective of their biological functions, their presence in spermatozoa can be taken advantage as biomarkers for sperm quality analysis. Investigations into the utility of sperm RNAs as biomarkers of fertility began in early 2000. In one of the earliest studies on gene expression in sperm, Patrizio et al. (2001) reported significant alterations in testicular gene expression in infertile patients (9). Later, sperm RNAs were found to echo gene expression in the testis in other studies (10). However, prior to using sperm RNAs as fertility biomarkers, the normal repertoire of sperm RNAs must be defined. It has been proposed that the ‘universal core’ of gene expression for spermatogenesis must be active, defining the normal transcriptome in spermatozoa. Nevertheless, the process of spermatogenesis is quite heterogeneous due to its quantitative nature, which may be further complicated by environmental factors. Therefore, carefully planned studies to identify the universal gene expression battery are critical to identifying RNA-based biomarkers of male fertility.

Microarray studies were hypothesized in the early 2000s to decipher spermatozoal RNA biomarkers. The first such study was conducted on Spanish men, which identified hundreds of genes to be differentially expressed (11). After this, a few other studies reported similar findings (12–14). We undertook a microarray study on sperm RNAs and found differential expressions of several RNAs in infertile spermatozoa (15). With the advent of next-generation transcriptome sequencing, a deeper investigation of transcriptome changes became possible. With time, other studies published in this regard showed that sperm RNAs were not randomly packed in these cells; instead, they displayed a consistent pattern. A study to analyze the consistency and quality of sperm RNAs revealed the high potential of these molecules to serve as biomarkers of sperm fertility (16). The utilization of sperm sncRNAs in assisted reproduction has also been investigated with significant success. For example, a comparison of sperm samples resulting in high and low rate of good quality embryos revealed significant differences in miRNA, tsRNA, and rsRNA profiles (17). They used PCA and support vector machine classifiers to identify the candidate sncRNAs that can be used as sperm quality biomarkers in assisted reproduction (17). This way, several microarray, qRT-PCR or sequencing studies between 2010 and 2022 investigated differential miRNA levels in sperm to identify biomarkers of fertility (18–25). We compared the data from these studies and identified a set of the most consistently differentially expressed miRNAs (26). These miRNAs were subjected to further in-depth analysis in a large sample size to critically evaluate their potential as biomarkers of sperm fertility (26). We found that hsa-miR-9-3p, hsa-miR-30b-5p and hsa-miR-122-5p had the highest potential as biomarkers of male fertility and sperm quality (25). However, this study investigated only seven miRNAs and several other miRNAs are potential candidates for such investigations.

## 4 What is the normal sperm transcriptome?

Irrespective of the past or futuristic roles of sperm-borne RNAs, their consistent pattern in spermatozoa signifies their suitability as biomarkers of sperm quality and fertility (26). As mentioned above, a comparison of the sperm-RNA repertoire in normozoospermic fertile individuals is required to define the normal sperm transcriptome. A few studies have attempted to identify universal small RNAs in normal human sperm. The earliest study in this direction identified 3,281 transcripts (mRNAs) in a pool of nine normozoospermic fertile men (10). Salas-Huetos et al., (2014) undertook a microarray study on ten donor samples received from normozoospermic fertile individuals and identified 221 universal miRNAs. The data presented in this study also emphasized huge variability in sperm RNAs, indicated by the detection of 452 miRNAs in some individuals (27). In a transcriptome sequencing study on three normozoospermic donors, Krawetz et al. (2011) identified miRNAs, piRNAs, and repeat-associated small RNAs that would be critical to post-fertilization development (2). In addition to similarities across individuals, these studies also emphasized on individual-specific nature of a large number of sncRNAs in human sperm. Despite the discovery of sperm RNAs about six decades ago, studies to define the normal sperm RNA repertoire have been limited. Lifestyle and diet, which differ significantly across individuals, have been suggested to affect sperm RNA composition significantly (28, 29), which poses another challenge to addressing the variability across samples.

## 5 Which RNAs are suitable as fertility markers?

A major question that remains unanswered is the type of RNAs that could be used as biomarkers of fertility. Among the RNAs present in spermatozoa, there are two choices: long RNAs (mRNAs and lncRNAs) and small RNAs (miRNAs, piRNAs, tsRNAs, rsRNAs, snRNAs, snoRNAs, mt-tRNAs, mt-rRNAs, and endo-siRNAs). Spermatozoa emerging from the testis are rich in miRNAs; however, during epididymal transit, spermatozoal small RNA profile gets transformed (4). While testicular small RNAs are more likely to be required for spermatogenesis, epididymal RNAs are more likely to serve functions in post-fertilization development. Interestingly, some reports on sperm RNAs have shown that long RNAs in spermatozoa may get degraded, resulting in the production of smaller RNAs, whose purpose and functions remain unknown (30, 31). Some studies published on sperm RNAs suggest that the breakdown of long RNAs into smaller RNAs is required to degrade the coding RNAs into smaller units, which helps the cell to get rid of what is not required. Other studies have emphasized the biological importance of small RNAs, suggesting that the small RNAs are generated to serve biological functions that are not served by the regular RNAs (32). It appears that apart from the presence of RNAs in spermatozoa, a number of unique phenomena with regard to RNA processing take place during germ cell maturation (33, 34). Further in-depth research is required to understand this dogma. Sequencing of complete transcripts using long-read sequencing in spermatozoa would answer many questions; however, such studies are scarce (35). Further, it is now well known that each mRNA is regulated by several miRNAs, and each small RNA may regulate several mRNAs post-transcriptionally. Interestingly, in the latter stages of spermatogenesis, i.e., beyond round spermatids, post-transcriptional regulation is suggested to play a major role as transcription is shut down (34). Therefore, regulation at the level of RNAs is more critical to successful sperm production. Since each small RNA can regulate several coding RNAs, we can substitute a large number of long RNA potential markers with a much smaller number of small RNAs. Research till date has largely focussed on mRNAs and miRNAs as biomarkers of fertility; nevertheless, there are several other small RNAs that await similar interrogation for their suitability as sperm quality biomarkers. Given the vast world of small RNAs and their significant presence in spermatozoa, we have not even touched the tip of the iceberg.

## 6 Sperm RNA integrity and covering sncRNAs

Assuming that some of the regular RNAs get biologically degraded to generate smaller transcripts, we may find a lot of fragmented RNAs in spermatozoa. These RNAs may be fragmented to different extents, which is not yet clear and is subject to further research in this area. Like other cells, sperm do not have intact ribosomal RNAs (31); therefore, the integrity of the isolated RNA is a major question in sperm RNA preparation. For small RNA sequencing in spermatozoa, libraries at present are prepared using standard kits where the first step is adaptor ligation, requiring 5′Phosphate and 3′OH, which at their best cover unmodified sncRNAs (e.g., miRNAs) and suffer from biased coverage of sncRNAs with modifications (e.g., piRNA and tsRNA). However, to cover the modified sncRNAs, new protocols have been developed, where the first step is RNA processing depending upon the type of modification in the target RNAs. These methods have been well-reviewed recently by Liu and Sharma (32). The implementation of the new protocols along with a good sample size which we have discussed in the later sections of this article are required to define the true picture of sperm RNAs. The identification of sperm RNA-based biomarkers needs focused research for the identification of small RNAs showing consistent expression, the optimization of protocol for the isolation of good quality RNAs, the determination of RNA quality for sperm samples, optimization of unbiased library preparation protocols, and the replication of data across independent cohorts (36, 37). The first landmark research in this aspect has to be undertaken on normozoospermic fertile samples looking for consistent expression of small RNAs in spermatozoa, followed by the comparison of the most consistent RNAs with infertile men, and possibly by a meta-analytical approach on all published data on small RNAs, which can take care of the issue of variations unrelated to fertility.

## 7 Sequencing depth and sample size requirements

With regard to gene expression study design on spermatozoa, two pertinent issues need consideration: sequencing depth and sample size. According to an estimate in gene expression studies, to cover 89% of genes, a sequencing depth of 25 million mapped reads is required, and to cover 100% of genes, a sequencing depth of 200 million is required; however, the gains for coverage beyond 10 million mapped reads are minimal (38). Therefore, we believe a sequencing depth of 10–25 million mapped reads is sufficient for gene expression studies. According to statistical estimates, to obtain a two-fold change in gene expression values at 80% power with alpha at 0.001 and a sequencing depth of 25 million mapped reads, the required sample size is highly dependent on the coefficient of variation (38). With a coefficient of variation of 0.4, 0.8, and 1.2, the required sample sizes would be about 6, 20, and 40 (38). In our experience of gene expression profile in human spermatozoa, expression values differ significantly from individual to individual and gene to gene, and the coefficient of variation for spermatozoal genes can be as high as 1.2, demanding sample sizes of up to 40 to detect a two-fold change in gene expression.

## 8 Sperm sncRNAs in transgenerational inheritance

In addition to acting as fertility biomarkers, sperm RNAs also mediate intergenerational and transgenerational inheritance, which have been suggested to have a significant impact on health. Various studies in rodents have demonstrated alterations in miRNAs, tsRNAs, and rsRNAs in response to environmental toxicants, chronic stress, diet, malnutrition, and obesity (29, 39–42). The most prominent study on this aspect was conducted by subjecting mice to trauma early in life (28). In this study, the newborn pups were separated from their mothers, followed by their behavioural analysis. The abnormal behaviour of the pups was interestingly inherited by the next generations as well, which correlated with alterations in miRNAs in the sperm and brain (28). Another interesting study reported that a stressful condition experienced by a parent can be transferred to the offspring through miRNAs (41). Nine miRNAs were increased in response to chronic stress in sperm and were associated with reduced hypothalamic-pituitary-adrenal (HPA) stress axis reactivity in offsprings. As a confirmatory test, these miRNAs, when injected *in vitro* into the zygote, showed a decreased cortisol level (41). There are other studies stating that experiences and stresses in life improve cognitive function and provide resilience (43); however, the exact mechanism of transgenerational/intergenerational inheritance remains unknown. Several early stressors and experiences in life have been shown to improve tolerability in the future. Another environmental factor that has been widely studied for epigenetic inheritance is the high-fat diet phenotype. Studies have shown that the abundance of miRNA let7c is altered in sperm in response to high fat (44) and a low-protein diet (42). In addition to miRNAs, the role of tRFs has been studied in intergenerational inheritance, particularly about diet-governed effects. Mice fed a low-protein diet gave birth to offsprings with altered lipid and cholesterol biosynthesis, and their sperm showed increased levels of tRFs derived from tRNAGlyGCC, which was functionally proven to regulate transcripts that were MERVL-driven, and many MERVL-driven genes are known to regulate zygotic genome activation (42, 45). Similarly, high-fat diet experiments with tRF alterations and their involvement in the transmittance of signals to the next generation were proven with the help of ICSI-derived embryos microinjected with 30–40 nt tRFs derived from high-fat sperm (29). Thus, apart from epigenetic changes, sperm RNAs (a non-genetic mechanism) could be central to such inheritance. It must be noted that these transgenerational effects have been demonstrated only in experimental animals and not in humans.

## 9 Conclusion

In a nutshell, the identification of sperm-quality RNA biomarkers is dependent on decoding the RNA repertoire of human spermatozoa, which needs to focus on small RNAs. Various laboratories have been looking for differences in gene expression since 2000, but the normal transcriptome of human spermatozoa has not yet been defined, which is a critical step in the identification of biomarkers of fertility. The identification of sperm RNA biomarkers would help in the selection of quality donor samples for assisted reproduction, explain infertility in idiopathic cases, and identify small RNAs for RNA therapeutics in infertility treatment. This would be helpful in predicting the chances of success and improving the success rate of assisted reproduction. Further, these RNAs can be used as health biomarkers for transgenerational inheritance of various exposures, which may or may not affect fertility.

## Data Availability

The original contributions presented in the study are included in the article/Supplementary Material, further inquiries can be directed to the corresponding author.

## References

[1] Abraham KA Bhargava PM. Nucleic acid metabolism of mammalian spermatozoa. Biochem J. (1963) 86(2):298. 10.1042/bj0860298 14010728 PMC1201753

[2] Krawetz SA Kruger A Lalancette C Tagett R Anton E Draghici S A survey of small RNAs in human sperm. Hum Reprod. (2011) 26(12):3401–12. 10.1093/humrep/der329 21989093 PMC3212879

[3] Miller D. RNA In the ejaculate spermatozoon: a window into molecular events in spermatogenesis and a record of the unusual requirements of haploid gene expression and post-meiotic equilibration. Mol Hum Reprod. (1997) 3(8):669–76. 10.1093/molehr/3.8.669 9294850

[4] Sharma U Sun F Conine CC Reichholf B Kukreja S Herzog VA Small RNAs are trafficked from the epididymis to developing mammalian sperm. Dev Cell. (2018) 46(4):481–94. 10.1016/j.devcel.2018.06.023 30057273 PMC6103849

[5] Miller D. Confrontation, consolidation, and recognition: the oocyte’s perspective on the incoming sperm. Cold Spring Harbor Perspect Med. (2015) 5(8):a023408. 10.1101/cshperspect.a023408 PMC4526728 25957313

[6] Liu TE Cheng W Gao Y Wang HU Liu Z. Microarray analysis of microRNA expression patterns in the semen of infertile men with semen abnormalities. Mol Med Rep. (2012) 6(3):535–42. 10.3892/mmr.2012.967 22735917

[7] Conine CC Sun F Song L Rivera-Pérez JA Rando OJ. Small RNAs gained during epididymal transit of sperm are essential for embryonic development in mice. Dev Cell. (2018) 46(4):470–80. 10.1016/j.devcel.2018.06.024 30057276 PMC6103825

[8] Yuan S Schuster A Tang C Yu T Ortogero N Bao J Sperm-borne miRNAs and endo-siRNAs are important for fertilization and preimplantation embryonic development. Development. (2016) 143(4):635–47. 10.1242/dev.131755 26718009 PMC4760322

[9] Patrizio P Hecht N Rockett J Schmid J Dix D. DNA Microarrays to study gene expression profiles in the testis of fertile and infertile men. Fertil Steril. (2001) 76(3):S40. 10.1016/S0015-0282(01)02136-7

[10] Ostermeier GC Dix DJ Miller D Khatri P Krawetz SA. Spermatozoal RNA profiles of normal fertile men. Lancet. (2002) 360(9335):772–7. 10.1016/S0140-6736(02)09899-9 12241836

[11] Garrido N Martinez-Conejero JA Jauregui J Horcajadas JA Simon C Remohi J Microarray analysis in sperm from fertile and infertile men without basic sperm analysis abnormalities reveals a significantly different transcriptome. Fertil Steril. (2009) 91(4):1307–10. 10.1016/j.fertnstert.2008.01.078 18367176

[12] Lalancette C Platts AE Johnson GD Emery BR Carrell DT Krawetz SA. Identification of human sperm transcripts as candidate markers of male fertility. J Mol Med. (2009) 87:735–48. 10.1007/s00109-009-0485-9 19466390

[13] García-Herrero S Meseguer M Martínez-Conejero JA Remohí J Pellicer A Garrido N. The transcriptome of spermatozoa used in homologous intrauterine insemination varies considerably between samples that achieve pregnancy and those that do not. Fertil Steril. (2010) 94(4):1360–73. 10.1016/j.fertnstert.2009.07.1671 19796764

[14] García-Herrero S Garrido N Martínez-Conejero JA Remohí J Pellicer A Meseguer M. Differential transcriptomic profile in spermatozoa achieving pregnancy or not via ICSI. Reprod Biomed Online. (2011) 22(1):25–36. 10.1016/j.rbmo.2010.09.013 21123116

[15] Bansal SK Gupta N Sankhwar SN Rajender S. Differential genes expression between fertile and infertile spermatozoa revealed by transcriptome analysis. PloS One. (2015) 10(5):e0127007. 10.1371/journal.pone.0127007 25973848 PMC4431685

[16] Georgiadis AP Kishore A Zorrilla M Jaffe TM Sanfilippo JS Volk E High-quality RNA in semen and sperm: isolation, analysis and potential application in clinical testing. J Urol. (2015) 193(1):352–9. 10.1016/j.juro.2014.07.107 25088949 PMC4382362

[17] Hua M Liu W Chen Y Zhang F Xu B Liu S Identification of small non-coding RNAs as sperm quality biomarkers for in vitro fertilization. Cell Discov. (2019) 5:20. 10.1038/s41421-019-0087-9 30992999 PMC6453904

[18] Liu WM Pang RT Chiu PC Wong BP Lao K Lee KF Sperm-borne microRNA-34c is required for the first cleavage division in mouse. Proc Natl Acad Sci USA. (2012) 109(2):490–4. 10.1073/pnas.1110368109 22203953 PMC3258645

[19] Abu-Halima M Hammadeh M Schmitt J Leidinger P Keller A Meese E Altered microRNA expression profiles of human spermatozoa in patients with different spermatogenic impairments. Fertil Steril. (2013) 99(5):1249–55. 10.1016/j.fertnstert.2012.11.054 23312218

[20] Abhari A Zarghami N Farzadi L Nouri M Shahnazi V. Altered of microRNA expression level in oligospermic patients. Iran J Reprod Med. (2014) 12(10):681. 25469126 PMC4248154

[21] Salas-Huetos A Blanco J Vidal F Godo A Grossmann M Pons MC Spermatozoa from patients with seminal alterations exhibit a differential micro-ribonucleic acid profile. Fertil Steril. (2015) 104(3):591–601. 10.1016/j.fertnstert.2015.06.015 26143365

[22] Muñoz X Mata A Bassas L Larriba S. Altered miRNA signature of developing germ-cells in infertile patients relates to the severity of spermatogenic failure and persists in spermatozoa. Sci Rep. (2015) 5(1):17991. 10.1038/srep17991 26648257 PMC4673613

[23] Salas-Huetos A Blanco J Vidal F Grossmann M Pons MC Garrido N Spermatozoa from normozoospermic fertile and infertile individuals convey a distinct mi RNA cargo. Andrology. (2016) 4(6):1028–36. 10.1111/andr.12276 27676136

[24] Mokánszki A Molnár Z Varga Tothne E Bodnár B Jakab A Bálint BL Altered microRNAs expression levels of sperm and seminal plasma in patients with infertile ejaculates compared with normozoospermic males. Hum Fertil. (2020) 23(4):246–55. 10.1080/14647273.2018.1562241 30632823

[25] Joshi M Andrabi SW Singh V Bansal SK Makker GC Mishra G Coding and regulatory transcriptome comparisons between fertile and infertile spermatozoa identify RNA signatures of male infertility. Andrologia. (2022) 54(7):e14437. 10.1111/and.14437 35437806

[26] Joshi M Andrabi SW Yadav RK Sankhwar SN Gupta G Rajender S. Qualitative and quantitative assessment of sperm miRNAs identifies hsa-miR-9-3p, hsa-miR-30b-5p and hsa-miR-122-5p as potential biomarkers of male infertility and sperm quality. Reprod Biol Endocrinol. (2022) 20(1):122. 10.1186/s12958-022-00990-7 35971175 PMC9377062

[27] Salas-Huetos A Blanco J Vidal F Mercader JM Garrido N Anton E. New insights into the expression profile and function of micro-ribonucleic acid in human spermatozoa. Fertil Steril. (2014) 102(1):213–22. 10.1016/j.fertnstert.2014.03.040 24794309

[28] Gapp K Jawaid A Sarkies P Bohacek J Pelczar P Prados J Implication of sperm RNAs in transgenerational inheritance of the effects of early trauma in mice. Nat Neurosci. (2014) 17(5):667–9. 10.1038/nn.3695 24728267 PMC4333222

[29] Chen Q Yan M Cao Z Li X Zhang Y Shi J Sperm tsRNAs contribute to intergenerational inheritance of an acquired metabolic disorder. Science. (2016) 351(6271):397–400. 10.1126/science.aad7977 26721680

[30] Gilbert I Bissonnette N Boissonneault G Vallée M Robert C. A molecular analysis of the population of mRNA in bovine spermatozoa. Reproduction. (2007) 133(6):1073–86. 10.1530/REP-06-0292 17636162

[31] Johnson GD Sendler E Lalancette C Hauser R Diamond MP Krawetz S. Cleavage of rRNA ensures translational cessation in sperm at fertilization. Mol Hum Reprod. (2011) 17(12):721–6. 10.1093/molehr/gar054 21831882 PMC3212859

[32] Liu S Sharma U. Sperm RNA payload: implications for intergenerational epigenetic inheritance. Int J Mol Sci. (2023) 24(6):5889. 10.3390/ijms24065889 36982962 PMC10052761

[33] Tang C Klukovich R Peng H Wang Z Yu T Zhang Y ALKBH5-dependent m6A demethylation controls splicing and stability of long 3'-UTR mRNAs in male germ cells. Proc Natl Acad Sci U S A. (2018) 115(2):E325–33. 10.1073/pnas.1717794115 29279410 PMC5777073

[34] Zhang Y Tang C Yu T Zhang R Zheng H Yan W. MicroRNAs control mRNA fate by compartmentalization based on 3' UTR length in male germ cells. Genome Biol. (2017) 18(1):105. 10.1186/s13059-017-1243-x 28615029 PMC5471846

[35] Sun YH Wang A Song C Shankar G Srivastava RK Au KF Single-molecule long-read sequencing reveals a conserved intact long RNA profile in sperm. Nat Commun. (2021) 12(1):1361. 10.1038/s41467-021-21524-6 33649327 PMC7921563

[36] Goodrich R Johnson G Krawetz SA. The preparation of human spermatozoal RNA for clinical analysis. Arch Androl. (2007) 53(3):161–7. 10.1080/01485010701216526 17612875

[37] Shtratnikova V Naumov V Bezuglov V Zheludkevich A Smigulina L Dikov Y Optimization of small RNA extraction and comparative study of NGS library preparation from low count sperm samples. Syst Biol Reprod Med. (2021) 67(3):230–43. 10.1080/19396368.2021.1912851 34082629

[38] Hart SN Therneau TM Zhang Y Poland GA Kocher JP. Calculating sample size estimates for RNA sequencing data. J Comput Biol. (2013) 20(12):970–8. 10.1089/cmb.2012.0283 23961961 PMC3842884

[39] Schuster A Skinner MK Yan W. Ancestral vinclozolin exposure alters the epigenetic transgenerational inheritance of sperm small noncoding RNAs. Environ Epigenet. (2016) 2(1):dvw001. 10.1093/eep/dvw001 27390623 PMC4933025

[40] Skinner MK Ben Maamar M Sadler-Riggleman I Beck D Nilsson E McBirney M Alterations in sperm DNA methylation, non-coding RNA and histone retention associate with DDT-induced epigenetic transgenerational inheritance of disease. Epigenetics Chromatin. (2018) 11(1):1–24. 10.1186/s13072-018-0178-0 29482626 PMC5827984

[41] Rodgers AB Morgan CP Leu NA Bale TL. Transgenerational epigenetic programming via sperm microRNA recapitulates effects of paternal stress. Proc Natl Acad Sci USA. (2015) 112(44):13699–704. 10.1073/pnas.1508347112 26483456 PMC4640733

[42] Sharma U Conine CC Shea JM Boskovic A Derr AG Bing XY Biogenesis and function of tRNA fragments during sperm maturation and fertilization in mammals. Science. (2016) 351(6271):391–6. 10.1126/science.aad6780 26721685 PMC4888079

[43] Hadar R Edemann-Callesen H Hlusicka EB Wieske F Vogel M Günther L Recurrent stress across life may improve cognitive performance in individual rats, suggesting the induction of resilience. Transl Psychiatry. (2019) 9(1):185. 10.1038/s41398-019-0523-5 31383851 PMC6683163

[44] de Castro Barbosa T Ingerslev LR Alm PS Versteyhe S Massart J Rasmussen M High-fat diet reprograms the epigenome of rat spermatozoa and transgenerationally affects metabolism of the offspring. Mol Metab. (2016) 5(3):184–97. 10.1016/j.molmet.2015.12.002 26977389 PMC4770269

[45] Macfarlan TS Gifford WD Driscoll S Lettieri K Rowe HM Bonanomi D Embryonic stem cell potency fluctuates with endogenous retrovirus activity. Nature. (2012) 487(7405):57–63. 10.1038/nature11244 22722858 PMC3395470

